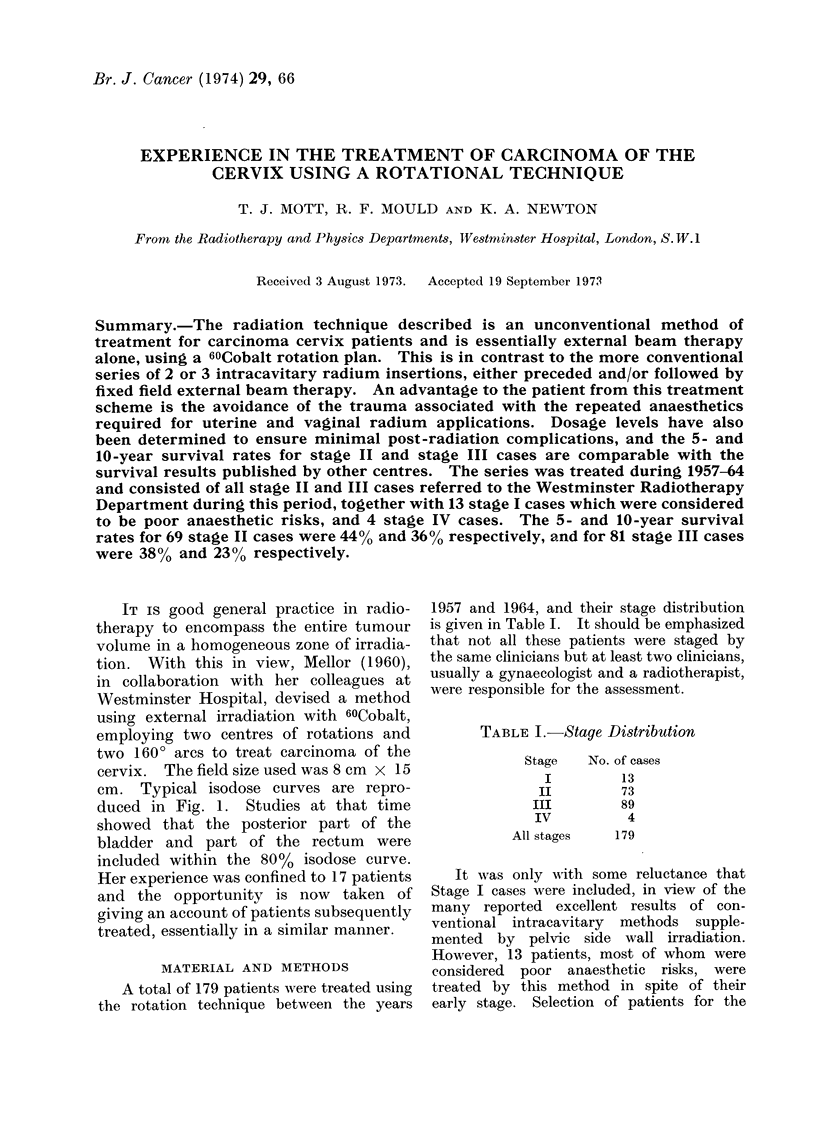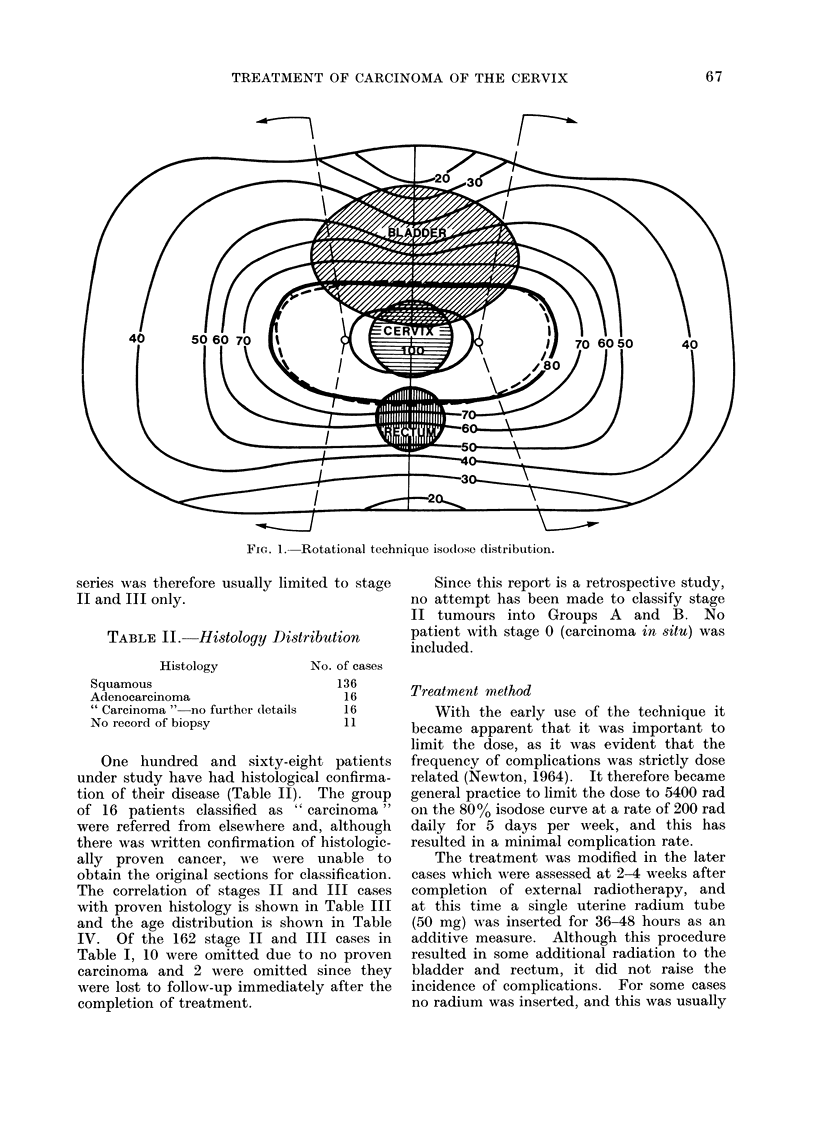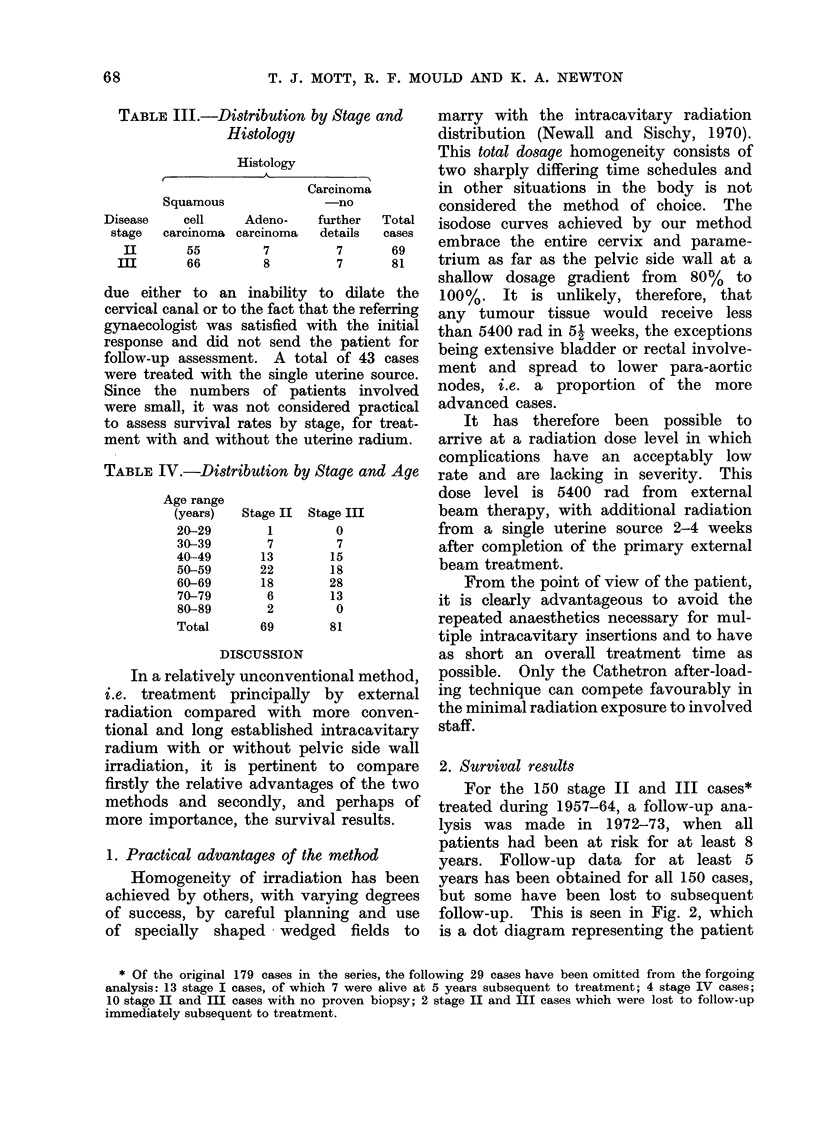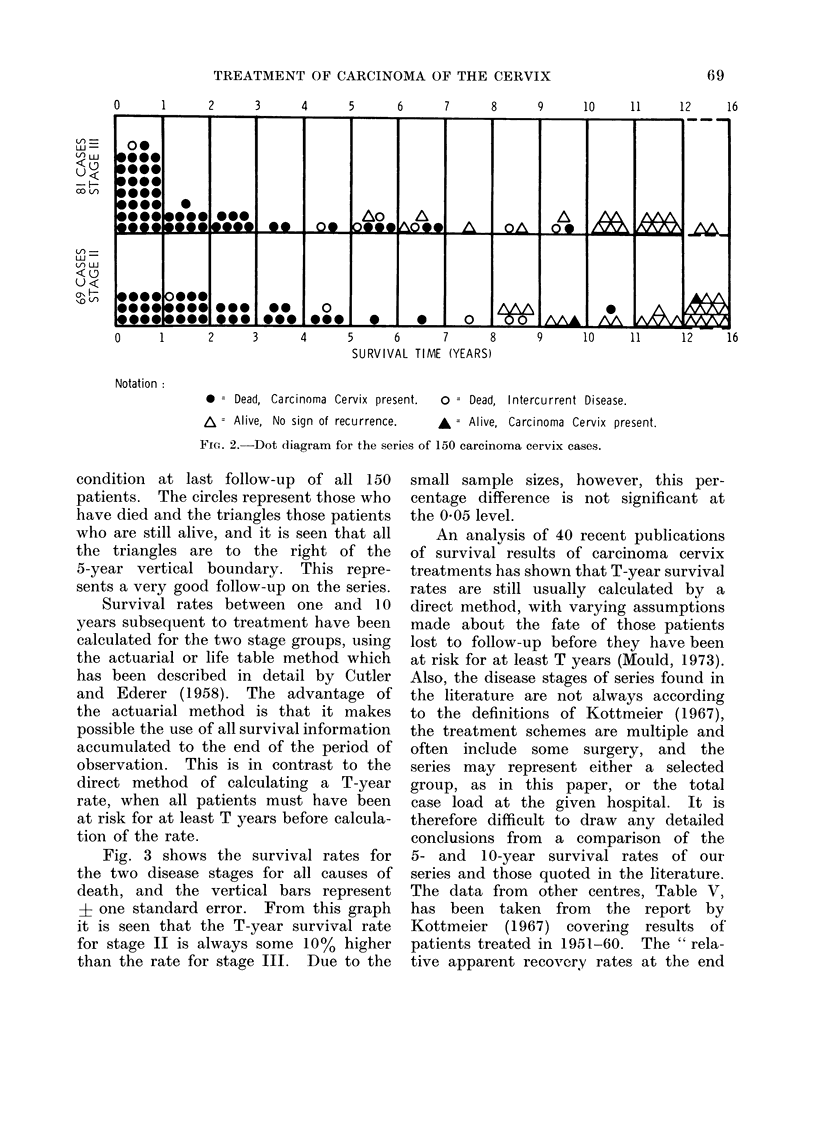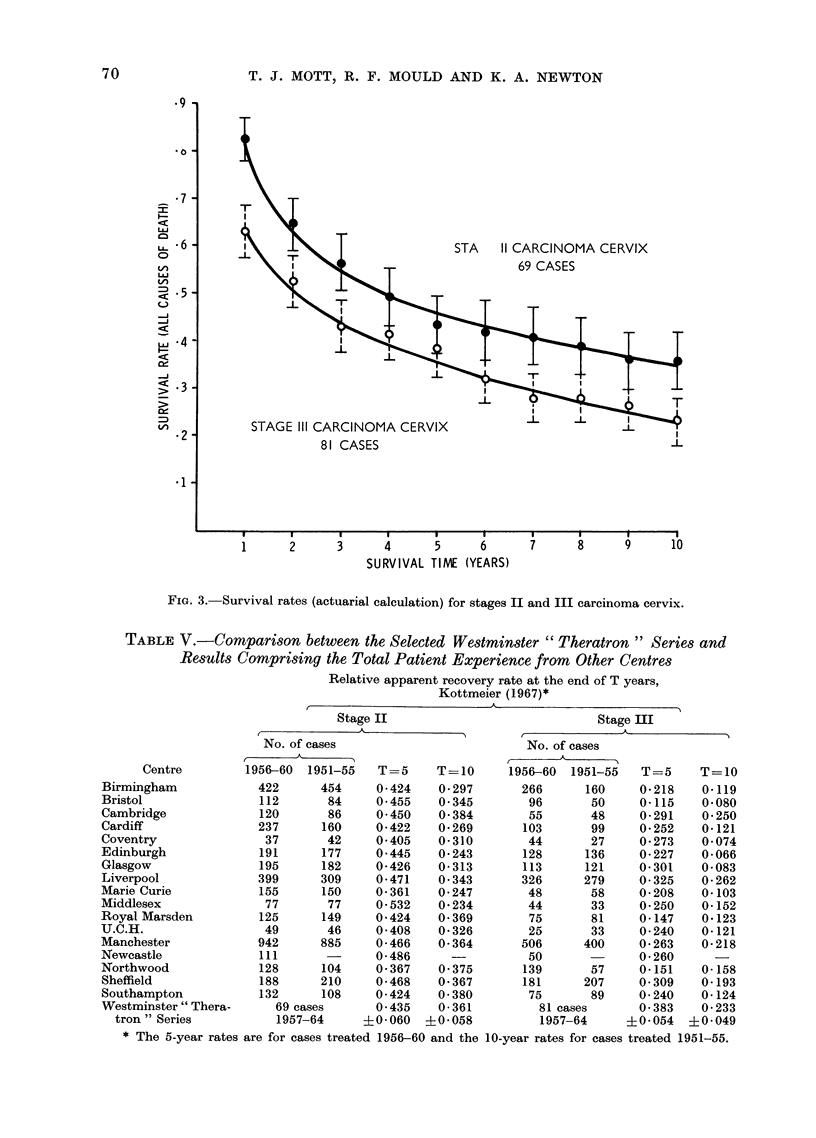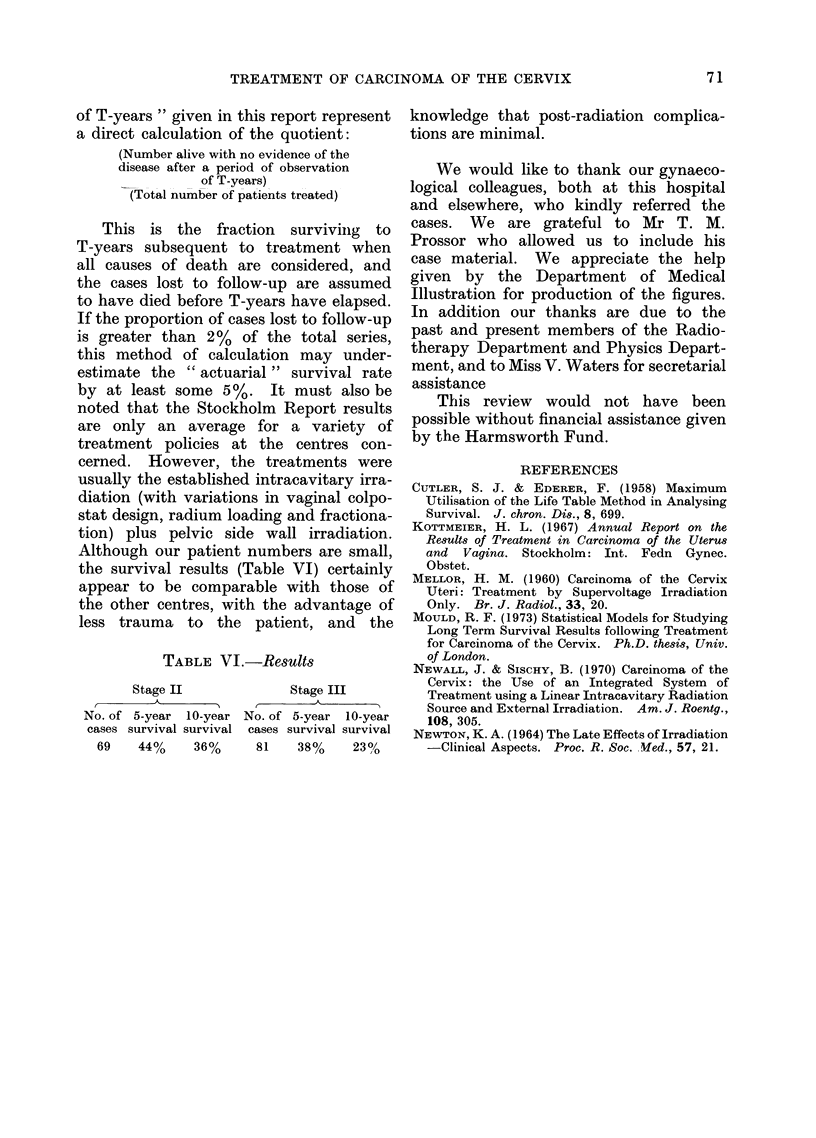# Experience in the Treatment of Carcinoma of the Cervix Using a Rotational Technique

**DOI:** 10.1038/bjc.1974.8

**Published:** 1974-01

**Authors:** T. J. Mott, R. F. Mould, K. A. Newton

## Abstract

The radiation technique described is an unconventional method of treatment for carcinoma cervix patients and is essentially external beam therapy alone, using a ^60^Cobalt rotation plan. This is in contrast to the more conventional series of 2 or 3 intracavitary radium insertions, either preceded and/or followed by fixed field external beam therapy. An advantage to the patient from this treatment scheme is the avoidance of the trauma associated with the repeated anaesthetics required for uterine and vaginal radium applications. Dosage levels have also been determined to ensure minimal post-radiation complications, and the 5- and 10-year survival rates for stage II and stage III cases are comparable with the survival results published by other centres. The series was treated during 1957-64 and consisted of all stage II and III cases referred to the Westminster Radiotherapy Department during this period, together with 13 stage I cases which were considered to be poor anaesthetic risks, and 4 stage IV cases. The 5- and 10-year survival rates for 69 stage II cases were 44% and 36% respectively, and for 81 stage III cases were 38% and 23% respectively.


*Br. J. Cancer* (1974) **29**, 66

### EXPERIENCE IN THE TREATMENT OF CARCINOMA OF THE CERVIX USING A ROTATIONAL TECHNIQUE

T. J. MOTT, R. F. MOULD AND K. A. NEWTON

*From the Radiotherapy and Physics Departments, Westminster Hospital, London, S.W.*1

Received 3 August 1973. Accepted 19 September 1973

**Summary.—The radiation technique described is an unconventional method of treatment for carcinoma cervix patients and is essentially external beam therapy alone, using a $^{60}$Cobalt rotation plan. This is in contrast to the more conventional series of 2 or 3 intracavitary radium insertions, either preceded and/or followed by fixed field external beam therapy. An advantage to the patient from this treatment scheme is the avoidance of the trauma associated with the repeated anaesthetics required for uterine and vaginal radium applications. Dosage levels have also been determined to ensure minimal post-radiation complications, and the 5- and 10-year survival rates for stage II and stage III cases are comparable with the survival results published by other centres. The series was treated during 1957–64 and consisted of all stage II and III cases referred to the Westminster Radiotherapy Department during this period, together with 13 stage I cases which were considered to be poor anaesthetic risks, and 4 stage IV cases. The 5- and 10-year survival rates for 69 stage II cases were 44% and 36% respectively, and for 81 stage III cases were 38% and 23% respectively.**

It is good general practice in radiotherapy to encompass the entire tumour volume in a homogeneous zone of irradiation. With this in view, Mellor (1960), in collaboration with her colleagues at Westminster Hospital, devised a method using external irradiation with $^{60}$Cobalt, employing two centres of rotations and two 160° arcs to treat carcinoma of the cervix. The field size used was 8 cm × 15 cm. Typical isodose curves are reproduced in Fig. 1. Studies at that time showed that the posterior part of the bladder and part of the rectum were included within the 80% isodose curve. Her experience was confined to 17 patients and the opportunity is now taken of giving an account of patients subsequently treated, essentially in a similar manner.

### MATERIAL AND METHODS

A total of 179 patients were treated using the rotation technique between the years 1957 and 1964, and their stage distribution is given in Table I. It should be emphasized that not all these patients were staged by the same clinicians but at least two clinicians, usually a gynaecologist and a radiotherapist, were responsible for the assessment.

TABLE I.—*Stage Distribution*

| Stage | No. of cases |
|---|---|
| I | 13 |
| II | 73 |
| III | 89 |
| IV | 4 |
| All stages | 179 |

It was only with some reluctance that Stage I cases were included, in view of the many reported excellent results of conventional intracavitary methods supplemented by pelvic side wall irradiation. However, 13 patients, most of whom were considered poor anaesthetic risks, were treated by this method in spite of their early stage. Selection of patients for the

FIG. 1.—Rotational technique isodose distribution.

series was therefore usually limited to stage II and III only.

TABLE II.—*Histology Distribution*

| Histology | No. of cases |
|---|---|
| Squamous | 136 |
| Adenocarcinoma | 16 |
| " Carcinoma "—no further details | 16 |
| No record of biopsy | 11 |

One hundred and sixty-eight patients under study have had histological confirmation of their disease (Table II). The group of 16 patients classified as " carcinoma " were referred from elsewhere and, although there was written confirmation of histologically proven cancer, we were unable to obtain the original sections for classification. The correlation of stages II and III cases with proven histology is shown in Table III and the age distribution is shown in Table IV. Of the 162 stage II and III cases in Table I, 10 were omitted due to no proven carcinoma and 2 were omitted since they were lost to follow-up immediately after the completion of treatment.

Since this report is a retrospective study, no attempt has been made to classify stage II tumours into Groups A and B. No patient with stage 0 (carcinoma *in situ*) was included.

*Treatment method*

With the early use of the technique it became apparent that it was important to limit the dose, as it was evident that the frequency of complications was strictly dose related (Newton, 1964). It therefore became general practice to limit the dose to 5400 rad on the 80% isodose curve at a rate of 200 rad daily for 5 days per week, and this has resulted in a minimal complication rate.

The treatment was modified in the later cases which were assessed at 2–4 weeks after completion of external radiotherapy, and at this time a single uterine radium tube (50 mg) was inserted for 36–48 hours as an additive measure. Although this procedure resulted in some additional radiation to the bladder and rectum, it did not raise the incidence of complications. For some cases no radium was inserted, and this was usually

TABLE III.—*Distribution by Stage and Histology*

| Disease stage | Histology: Squamous cell carcinoma | Adeno-carcinoma | Carcinoma—no further details | Total cases |
|---|---|---|---|---|
| II | 55 | 7 | 7 | 69 |
| III | 66 | 8 | 7 | 81 |

due either to an inability to dilate the cervical canal or to the fact that the referring gynaecologist was satisfied with the initial response and did not send the patient for follow-up assessment. A total of 43 cases were treated with the single uterine source. Since the numbers of patients involved were small, it was not considered practical to assess survival rates by stage, for treatment with and without the uterine radium.

TABLE IV.—*Distribution by Stage and Age*

| Age range (years) | Stage II | Stage III |
|---|---|---|
| 20–29 | 1 | 0 |
| 30–39 | 7 | 7 |
| 40–49 | 13 | 15 |
| 50–59 | 22 | 18 |
| 60–69 | 18 | 28 |
| 70–79 | 6 | 13 |
| 80–89 | 2 | 0 |
| Total | 69 | 81 |

### DISCUSSION

In a relatively unconventional method, *i.e.* treatment principally by external radiation compared with more conventional and long established intracavitary radium with or without pelvic side wall irradiation, it is pertinent to compare firstly the relative advantages of the two methods and secondly, and perhaps of more importance, the survival results.

### 1. *Practical advantages of the method*

Homogeneity of irradiation has been achieved by others, with varying degrees of success, by careful planning and use of specially shaped wedged fields to marry with the intracavitary radiation distribution (Newall and Sischy, 1970). This *total dosage* homogeneity consists of two sharply differing time schedules and in other situations in the body is not considered the method of choice. The isodose curves achieved by our method embrace the entire cervix and parametrium as far as the pelvic side wall at a shallow dosage gradient from 80% to 100%. It is unlikely, therefore, that any tumour tissue would receive less than 5400 rad in $5\frac{1}{2}$ weeks, the exceptions being extensive bladder or rectal involvement and spread to lower para-aortic nodes, *i.e.* a proportion of the more advanced cases.

It has therefore been possible to arrive at a radiation dose level in which complications have an acceptably low rate and are lacking in severity. This dose level is 5400 rad from external beam therapy, with additional radiation from a single uterine source 2–4 weeks after completion of the primary external beam treatment.

From the point of view of the patient, it is clearly advantageous to avoid the repeated anaesthetics necessary for multiple intracavitary insertions and to have as short an overall treatment time as possible. Only the Cathetron after-loading technique can compete favourably in the minimal radiation exposure to involved staff.

### 2. *Survival results*

For the 150 stage II and III cases* treated during 1957–64, a follow-up analysis was made in 1972–73, when all patients had been at risk for at least 8 years. Follow-up data for at least 5 years has been obtained for all 150 cases, but some have been lost to subsequent follow-up. This is seen in Fig. 2, which is a dot diagram representing the patient

* Of the original 179 cases in the series, the following 29 cases have been omitted from the forgoing analysis: 13 stage I cases, of which 7 were alive at 5 years subsequent to treatment; 4 stage IV cases; 10 stage II and III cases with no proven biopsy; 2 stage II and III cases which were lost to follow-up immediately subsequent to treatment.

FIG. 2.—Dot diagram for the series of 150 carcinoma cervix cases.

condition at last follow-up of all 150 patients. The circles represent those who have died and the triangles those patients who are still alive, and it is seen that all the triangles are to the right of the 5-year vertical boundary. This represents a very good follow-up on the series.

Survival rates between one and 10 years subsequent to treatment have been calculated for the two stage groups, using the actuarial or life table method which has been described in detail by Cutler and Ederer (1958). The advantage of the actuarial method is that it makes possible the use of all survival information accumulated to the end of the period of observation. This is in contrast to the direct method of calculating a T-year rate, when all patients must have been at risk for at least T years before calculation of the rate.

Fig. 3 shows the survival rates for the two disease stages for all causes of death, and the vertical bars represent $\pm$ one standard error. From this graph it is seen that the T-year survival rate for stage II is always some 10% higher than the rate for stage III. Due to the small sample sizes, however, this percentage difference is not significant at the 0·05 level.

An analysis of 40 recent publications of survival results of carcinoma cervix treatments has shown that T-year survival rates are still usually calculated by a direct method, with varying assumptions made about the fate of those patients lost to follow-up before they have been at risk for at least T years (Mould, 1973). Also, the disease stages of series found in the literature are not always according to the definitions of Kottmeier (1967), the treatment schemes are multiple and often include some surgery, and the series may represent either a selected group, as in this paper, or the total case load at the given hospital. It is therefore difficult to draw any detailed conclusions from a comparison of the 5- and 10-year survival rates of our series and those quoted in the literature. The data from other centres, Table V, has been taken from the report by Kottmeier (1967) covering results of patients treated in 1951–60. The "relative apparent recovery rates at the end

FIG. 3.—Survival rates (actuarial calculation) for stages II and III carcinoma cervix.

TABLE V.—*Comparison between the Selected Westminster "Theratron" Series and Results Comprising the Total Patient Experience from Other Centres*

| | Relative apparent recovery rate at the end of T years, Kottmeier (1967)* | | | | | | | |
|---|---|---|---|---|---|---|---|---|
| | Stage II | | | | Stage III | | | |
| | No. of cases | | | | No. of cases | | | |
| Centre | 1956–60 | 1951–55 | T=5 | T=10 | 1956–60 | 1951–55 | T=5 | T=10 |
| Birmingham | 422 | 454 | 0·424 | 0·297 | 266 | 160 | 0·218 | 0·119 |
| Bristol | 112 | 84 | 0·455 | 0·345 | 96 | 50 | 0·115 | 0·080 |
| Cambridge | 120 | 86 | 0·450 | 0·384 | 55 | 48 | 0·291 | 0·250 |
| Cardiff | 237 | 160 | 0·422 | 0·269 | 103 | 99 | 0·252 | 0·121 |
| Coventry | 37 | 42 | 0·405 | 0·310 | 44 | 27 | 0·273 | 0·074 |
| Edinburgh | 191 | 177 | 0·445 | 0·243 | 128 | 136 | 0·227 | 0·066 |
| Glasgow | 195 | 182 | 0·426 | 0·313 | 113 | 121 | 0·301 | 0·083 |
| Liverpool | 399 | 309 | 0·471 | 0·343 | 326 | 279 | 0·325 | 0·262 |
| Marie Curie | 155 | 150 | 0·361 | 0·247 | 48 | 58 | 0·208 | 0·103 |
| Middlesex | 77 | 77 | 0·532 | 0·234 | 44 | 33 | 0·250 | 0·152 |
| Royal Marsden | 125 | 149 | 0·424 | 0·369 | 75 | 81 | 0·147 | 0·123 |
| U.C.H. | 49 | 46 | 0·408 | 0·326 | 25 | 33 | 0·240 | 0·121 |
| Manchester | 942 | 885 | 0·466 | 0·364 | 506 | 400 | 0·263 | 0·218 |
| Newcastle | 111 | — | 0·486 | — | 50 | — | 0·260 | — |
| Northwood | 128 | 104 | 0·367 | 0·375 | 139 | 57 | 0·151 | 0·158 |
| Sheffield | 188 | 210 | 0·468 | 0·367 | 181 | 207 | 0·309 | 0·193 |
| Southampton | 132 | 108 | 0·424 | 0·380 | 75 | 89 | 0·240 | 0·124 |
| Westminster "Thera- | 69 cases | | 0·435 | 0·361 | 81 cases | | 0·383 | 0·233 |
| tron" Series | 1957–64 | | ±0·060 | ±0·058 | 1957–64 | | ±0·054 | ±0·049 |

* The 5-year rates are for cases treated 1956–60 and the 10-year rates for cases treated 1951–55.

of T-years " given in this report represent a direct calculation of the quotient:

$$\frac{\text{(Number alive with no evidence of the disease after a period of observation of T-years)}}{\text{(Total number of patients treated)}}$$

This is the fraction surviving to T-years subsequent to treatment when all causes of death are considered, and the cases lost to follow-up are assumed to have died before T-years have elapsed. If the proportion of cases lost to follow-up is greater than 2% of the total series, this method of calculation may underestimate the " actuarial " survival rate by at least some 5%. It must also be noted that the Stockholm Report results are only an average for a variety of treatment policies at the centres concerned. However, the treatments were usually the established intracavitary irradiation (with variations in vaginal colpostat design, radium loading and fractionation) plus pelvic side wall irradiation. Although our patient numbers are small, the survival results (Table VI) certainly appear to be comparable with those of the other centres, with the advantage of less trauma to the patient, and the knowledge that post-radiation complications are minimal.

TABLE VI.—*Results*

| Stage II | | | Stage III | | |
|---|---|---|---|---|---|
| No. of cases | 5-year survival | 10-year survival | No. of cases | 5-year survival | 10-year survival |
| 69 | 44% | 36% | 81 | 38% | 23% |

We would like to thank our gynaecological colleagues, both at this hospital and elsewhere, who kindly referred the cases. We are grateful to Mr T. M. Prossor who allowed us to include his case material. We appreciate the help given by the Department of Medical Illustration for production of the figures. In addition our thanks are due to the past and present members of the Radiotherapy Department and Physics Department, and to Miss V. Waters for secretarial assistance

This review would not have been possible without financial assistance given by the Harmsworth Fund.

REFERENCES

CUTLER, S. J. & EDERER, F. (1958) Maximum Utilisation of the Life Table Method in Analysing Survival. *J. chron. Dis.*, **8**, 699.

KOTTMEIER, H. L. (1967) *Annual Report on the Results of Treatment in Carcinoma of the Uterus and Vagina.* Stockholm: Int. Fedn Gynec. Obstet.

MELLOR, H. M. (1960) Carcinoma of the Cervix Uteri: Treatment by Supervoltage Irradiation Only. *Br. J. Radiol.*, **33**, 20.

MOULD, R. F. (1973) Statistical Models for Studying Long Term Survival Results following Treatment for Carcinoma of the Cervix. *Ph.D. thesis, Univ. of London.*

NEWALL, J. & SISCHY, B. (1970) Carcinoma of the Cervix: the Use of an Integrated System of Treatment using a Linear Intracavitary Radiation Source and External Irradiation. *Am. J. Roentg.*, **108**, 305.

NEWTON, K. A. (1964) The Late Effects of Irradiation —Clinical Aspects. *Proc. R. Soc. Med.*, **57**, 21.