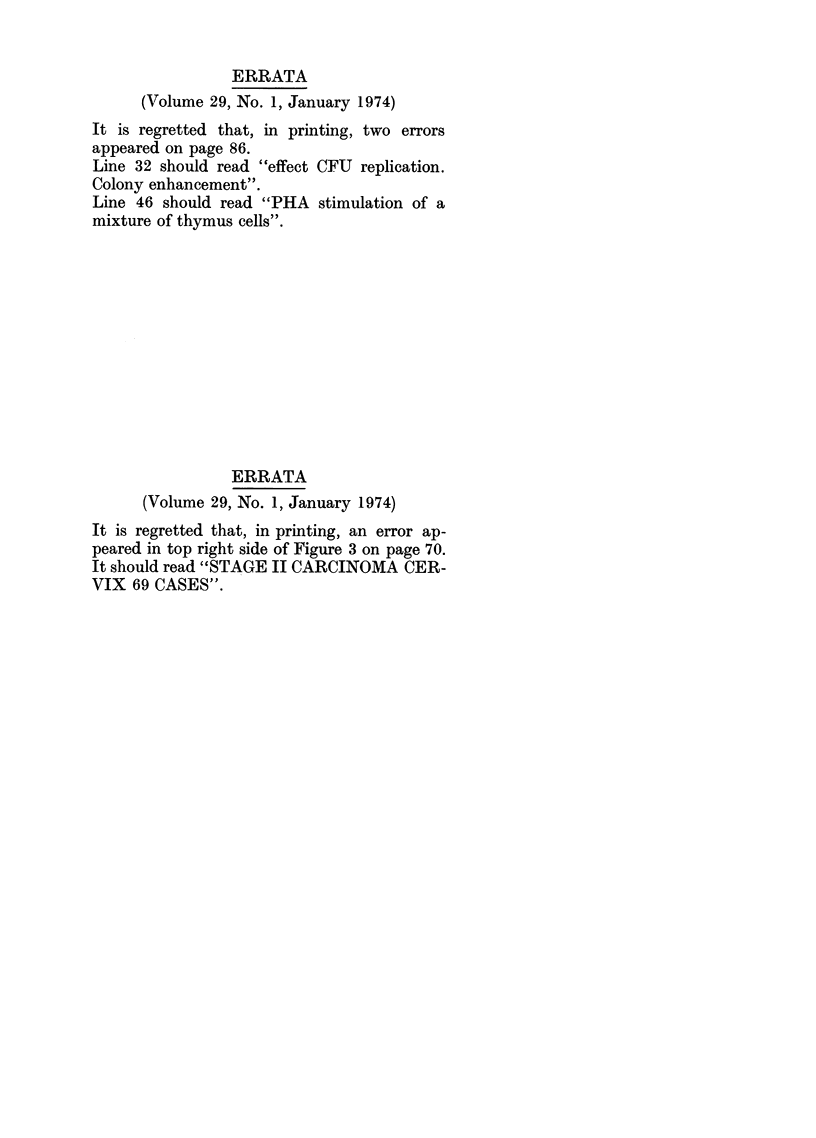# Errata

**Published:** 1974-01

**Authors:** 


					
ERRATA

(Volume 29, No. 1, January 1974)

It is regretted that, in printing, an error ap-
peared in top right side of Figure 3 on page 70.
It should read "STAGE II CARCINOMA CER-
VIX 69 CASES".